# DeepNPA: A protBert-based framework for residue-level annotation of neuropeptide precursors

**DOI:** 10.1016/j.isci.2026.117680

**Published:** 2026-09-29

**Authors:** Xin-Yu Li, Yang Yu, Li-Ping Liu, Meng-Ze Du, Qiang Tang, Juan-Juan Kang

**Affiliations:** 1 School of Intelligent Medicine, Innovative Institute of Chinese Medicine and Pharmacy, Chengdu University of Traditional Chinese Medicine, Chengdu 611137, China; 2 State Key Laboratory of Southwestern Chinese Medicine Resources, Chengdu University of Traditional Chinese Medicine, Chengdu 611137, China; 3 School of Healthcare Technology, Chengdu Neusoft University, Chengdu 611844, China; 4 School of Life Science and Technology, University of Electronic Science and Technology of China, Chengdu 611731, China; 5 Institute of Digestive Disease, the Affiliated Qingyuan Hospital (Qingyuan People’s Hospital), Guangzhou Medical University, Qingyuan 511518, China; 6 Key Laboratory of Non-coding RNA and Drug Discovery at Chengdu Medical College of Sichuan Province, School of Basic Medical Sciences, Chengdu Medical College, Chengdu 610500, China

**Keywords:** neuropeptide precursor, cleavage site prediction, residue-level annotation, ProtBert, focal loss

## Abstract

Neuropeptides are key signaling molecules that regulate the nervous and endocrine systems, and their maturation depends on precise proteolytic cleavage of precursor proteins. However, residue-level annotation remains essential yet experimentally labor intensive, with limited dedicated computational tools. Here, we present DeepNPA, a residue-level annotation framework that integrates ProtBert, a pretrained protein language model, with focal loss for imbalance-aware optimization. DeepNPA was trained and evaluated on a non-redundant Swiss-Prot-derived dataset annotated using the BIO scheme. On a held-out test dataset, DeepNPA achieved strong performance, with an accuracy of 0.953, an F1-score of 0.927, and an AUPRC of 0.965. On an independent benchmark dataset, DeepNPA outperformed NeuroCS and NeuroPred, achieving an ACC of 0.969 and Matthews correlation coefficient (MCC) of 0.666. External validation further demonstrated its ability to identify neuropeptide regions and their associated cleavage sites. In addition, we provided an online prediction platform to facilitate neuropeptide precursor annotation and cleavage site analysis.

## Highlights

• DeepNPA enables residue-level annotation of neuropeptide precursors
• Protein language models improve neuropeptide cleavage site prediction
• Focal loss enhances prediction of rare neuropeptide processing sites
• DeepNPA provides an online platform for neuropeptide precursor analysis

## Graphical abstract

**Figure:**
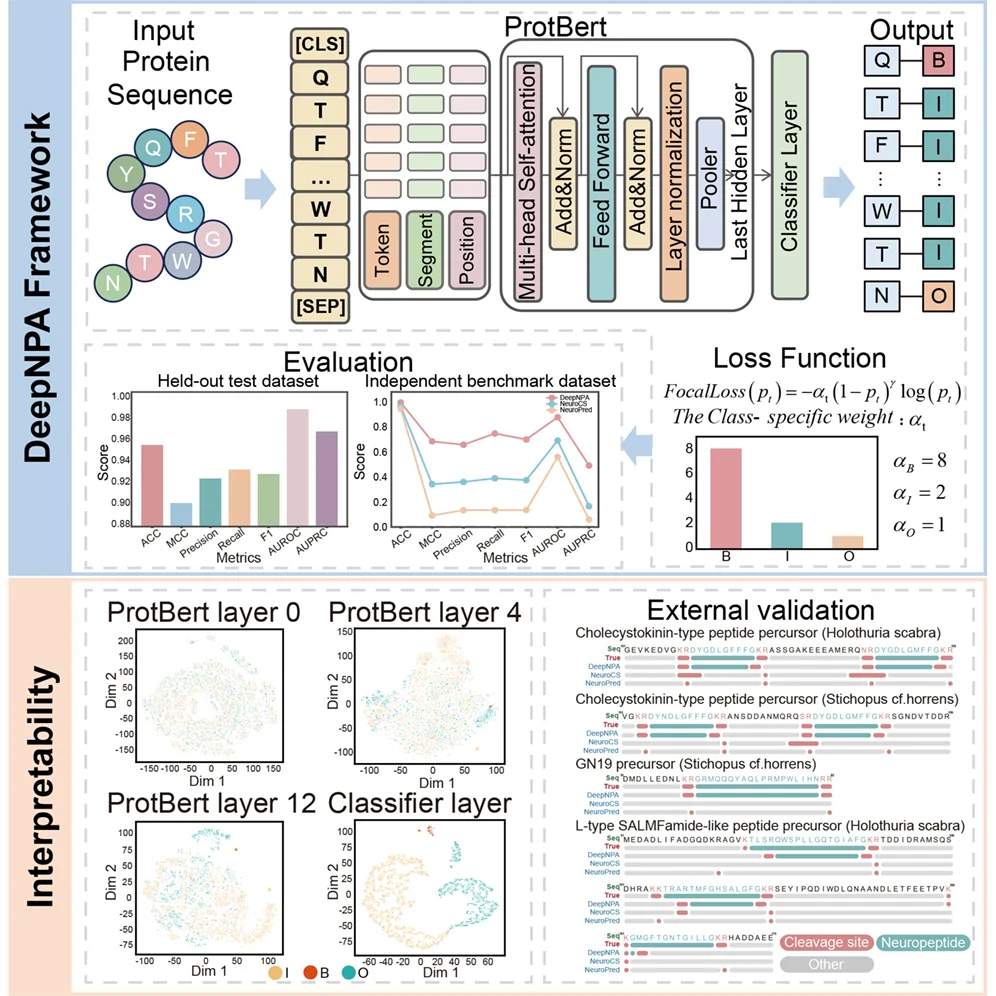

## Teaser

Classification of proteins; Bioinformatics; Artificial intelligence; Machine learning

## Introduction

Neuropeptides are pivotal mediators of intercellular communication, orchestrating a wide array of biological processes such as neuromodulation, metabolism, and immune regulation. These bioactive molecules are synthesized from larger neuropeptide precursors (NPPs) through highly specific and tightly regulated proteolytic cleavage events.^1^^,^^2^^,^^3^

Primarily acting through G protein-coupled receptors (GPCRs), neuropeptides modulate diverse physiological functions including nociception, energy homeostasis, emotional behavior, and cognitive processes.^4^^,^^5^^,^^6^ Owing to their central roles in physiological regulation and disease, neuropeptides have also attracted increasing attention as potential therapeutic targets in neurological and metabolic disorders. Accurate identification and annotation of neuropeptides are therefore essential for elucidating endogenous signaling mechanisms and facilitating neuropeptide-related functional studies.

However, experimental identification of neuropeptides and their cleavage patterns remain labor intensive, time consuming, and technically demanding. Despite the growing interest in neuropeptidomics, current computational efforts predominantly focus on two distinct tasks: identifying neuropeptide precursors or predicting their proteolytic cleavage sites. The former aims to distinguish neuropeptide precursors from other protein sequences using global features, while the latter focuses on localizing precise cleavage positions within annotated precursors. For example, in our earlier work, we developed NeuroPP, a machine learning-based tool that employs optimized amino acid composition features to identify neuropeptide precursors with high accuracy.^7^ Building upon this foundation, we subsequently introduced NeuroCS, which focuses on the prediction of cleavage sites within neuropeptide precursors using enhanced feature engineering and support vector machine classifiers.^8^ Another widely used framework for prohormone cleavage site prediction is NeuroPred, which originally implemented statistical and motif-based prediction models and has subsequently evolved to incorporate machine learning and, more recently, AI-based approaches.^9^^,^^10^ While these tools have advanced the field, dedicated computational tools capable of directly assigning residue-level tags to precursor sequences remain limited. Such residue-level annotation is important because it enables not only the identification of cleavage boundaries but also the delineation of neuropeptide regions within precursor proteins, thereby providing a more comprehensive view of neuropeptide maturation. However, severe class imbalance is inherent in neuropeptide precursors. Cleavage sites represent a minor fraction of residues compared to the overwhelming majority of non-cleavage sites, which can bias models toward the dominant class and reduce their sensitivity in detecting true cleavage positions.^11^

To address these limitations, we propose DeepNPA, a framework for residue-level annotation of neuropeptide precursors. DeepNPA integrates ProtBert, a protein language model pretrained on large-scale protein sequence corpora,^12^ with focal loss (FL) to mitigate class imbalance by emphasizing hard-to-classify instances.^13^ ProtBert provides deep contextualized embeddings that capture biochemical and evolutionary information, enhancing feature extraction. FL down-weights well-classified residues while focusing learning on underrepresented cleavage sites.

We systematically evaluated DeepNPA across diverse hyperparameter configurations and benchmarked its performance against NeuroCS and NeuroPred. DeepNPA achieved robust predictive performance and consistently outperformed existing methods. External validation further demonstrated that DeepNPA can effectively delineate neuropeptide regions within neuropeptide precursors. Finally, we developed a user-friendly online platform (https://i-health.info/DeepNPA/), thereby serving as a valuable resource for neuropeptide precursor annotation, cleavage site analysis, and downstream neuropeptidomics research.

## Results

### Dataset statistics

We collected manually reviewed neuropeptide precursor sequences and curated positional annotations of mature neuropeptide regions from the UniProtKB/Swiss-Prot database.^14^ Based on this positional information, we performed sequence labeling using the classical BIO (begin-inside-other) tagging scheme.^15^ Specifically, tag B denotes the start of a neuropeptide segment, I represents internal residues within a neuropeptide, and O corresponds to non-neuropeptide regions of the precursor (Figure 1A).

**Figure 1:**
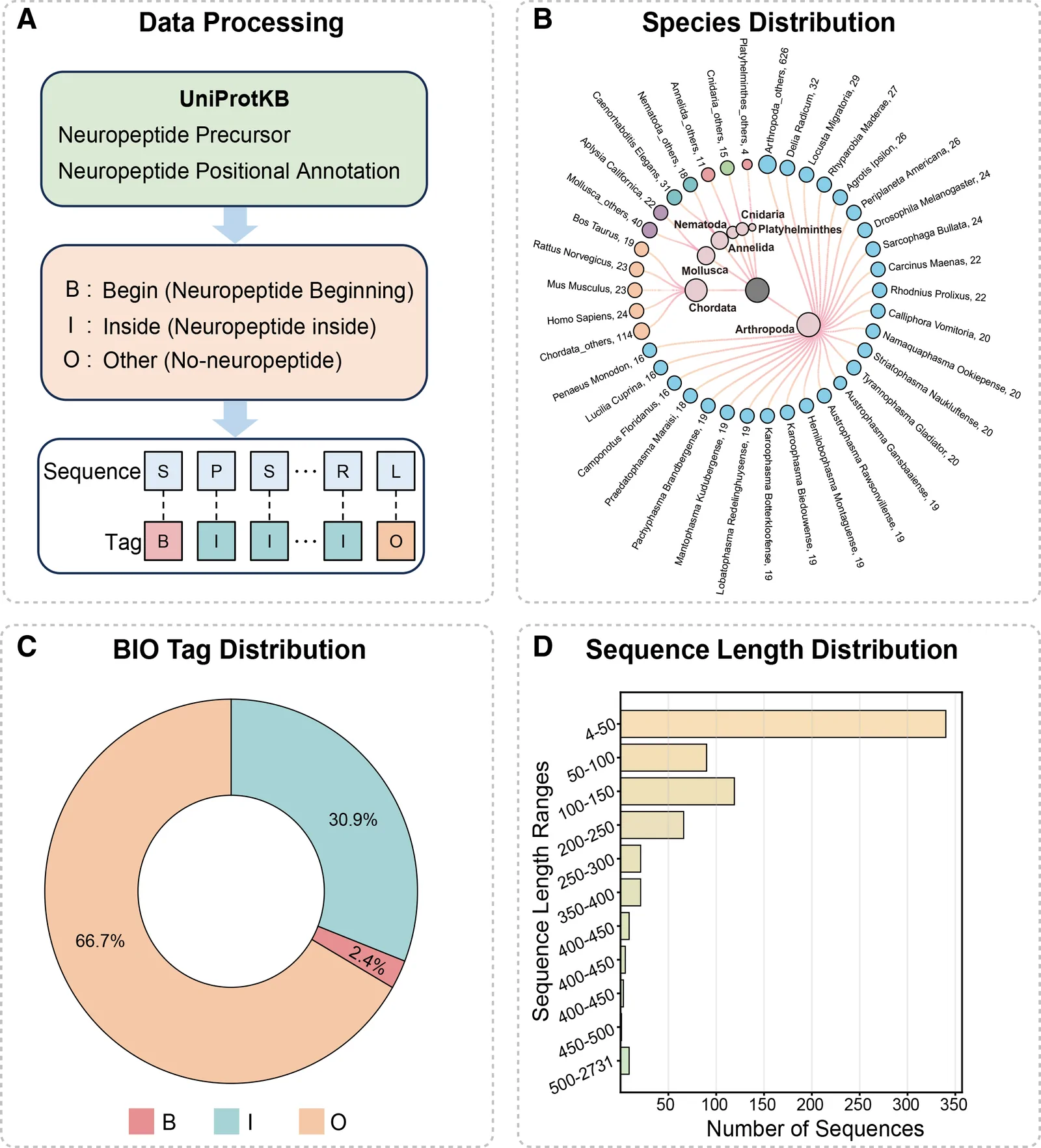
Overview of the neuropeptide precursor sequence annotation framework (A) Data preprocessing pipeline. (B) Species distribution of neuropeptide precursor sequences. (C) Class distribution of BIO tags. (D) Sequence length distribution.

In total, 1,500 neuropeptide precursor sequences were obtained from 273 species across 7 phyla, including Arthropoda, Chordata, Nematoda, Mollusca, Annelida, Platyhelminthes, and Cnidaria. Representative model organisms, including *Caenorhabditis elegans*, *Drosophila melanogaster*, *Homo sapiens*, *Mus musculus*, and *Rattus norvegicus*, were included (Figure 1B). The labeled dataset contains 1,577 B tags, 20,052 I tags, and 43,231 O tags, demonstrating a highly imbalanced residue distribution, with O tags accounting for 66.65% of all residues (Figure 1C). Additionally, the lengths of neuropeptide precursor sequences range from 4 to 2,731 amino acids, with the majority of sequences distributed between 4 and 350 residues (Figure 1D), reflecting the extensive structural diversity of neuropeptide precursor proteins.

### Overview of the DeepNPA framework

DeepNPA consists of three components: a ProtBert encoder for contextual representation learning, a linear classification head for BIO tag prediction, and a FL-based optimization objective designed to mitigate severe class imbalance (Figure 2). Given an input protein sequence, amino acid residues are tokenized using the standard ProtBert tokenizer. A classification token (CLS) is prepended to the sequence and a separator token (SEP) is appended to the end, following the original ProtBert specification. The tokenized sequence is mapped to embedding vectors and processed in parallel by the ProtBert encoder. Unlike autoregressive models, ProtBert employs bidirectional attention without causal masking; therefore, it generates position-aligned residue representations. As a result, DeepNPA formulates cleavage site prediction as a token-level classification task and does not require an explicit decoding layer. The contextualized hidden states from the final encoder layer are then passed to a linear classifier to produce logits over the BIO tag set.

**Figure 2:**
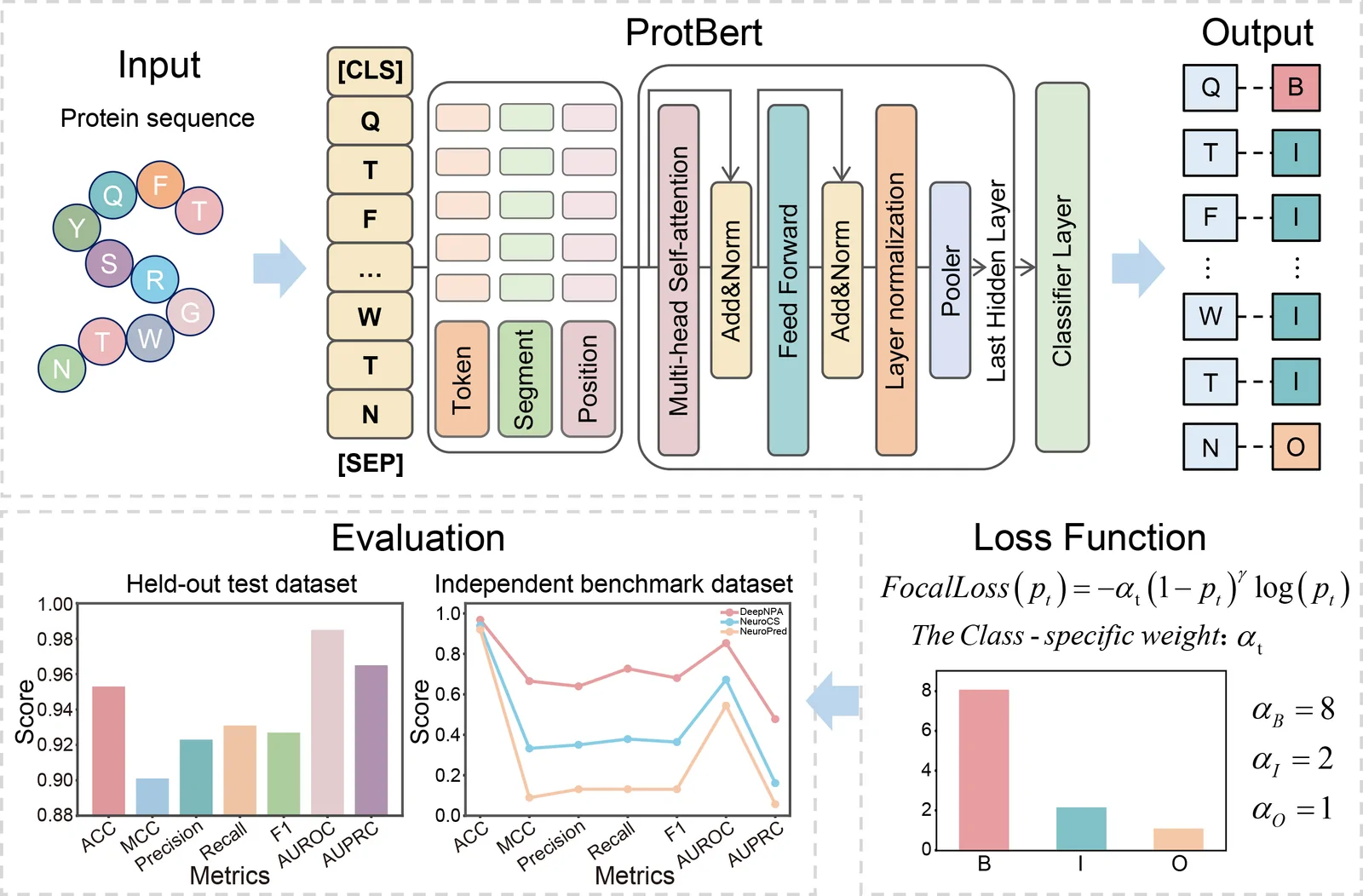
Architecture of the proposed annotation model

### Performance of DeepNPA on the held-out test dataset

DeepNPA demonstrated strong predictive performance on the held-out test dataset (Table 1), achieving consistent results across multiple evaluation metrics (Figure 3). Specifically, as shown in Figure 3A, the model attained an accuracy of 0.953, precision of 0.923, recall of 0.931, and an F1-score of 0.927, indicating balanced performance across different classes. Additionally, the area under the precision-recall curve (AUPRC) reached 0.965, and the area under the receiver operating characteristic curve (AUROC) achieved 0.985, underscoring DeepNPA’s excellent discriminative power and reliability, even in the presence of class imbalance.

**Table 1:** Summary of datasets

Dataset	Sequences	BIO tags		
		B	I	O
Train	479	1,094	13,850	32,212
Validation	68	159	1,931	3,191
Held-out test	138	329	4,271	7,828
Independent benchmark	50	189	2,509	5,127
External validation	16	60	818	1,899

**Figure 3:**
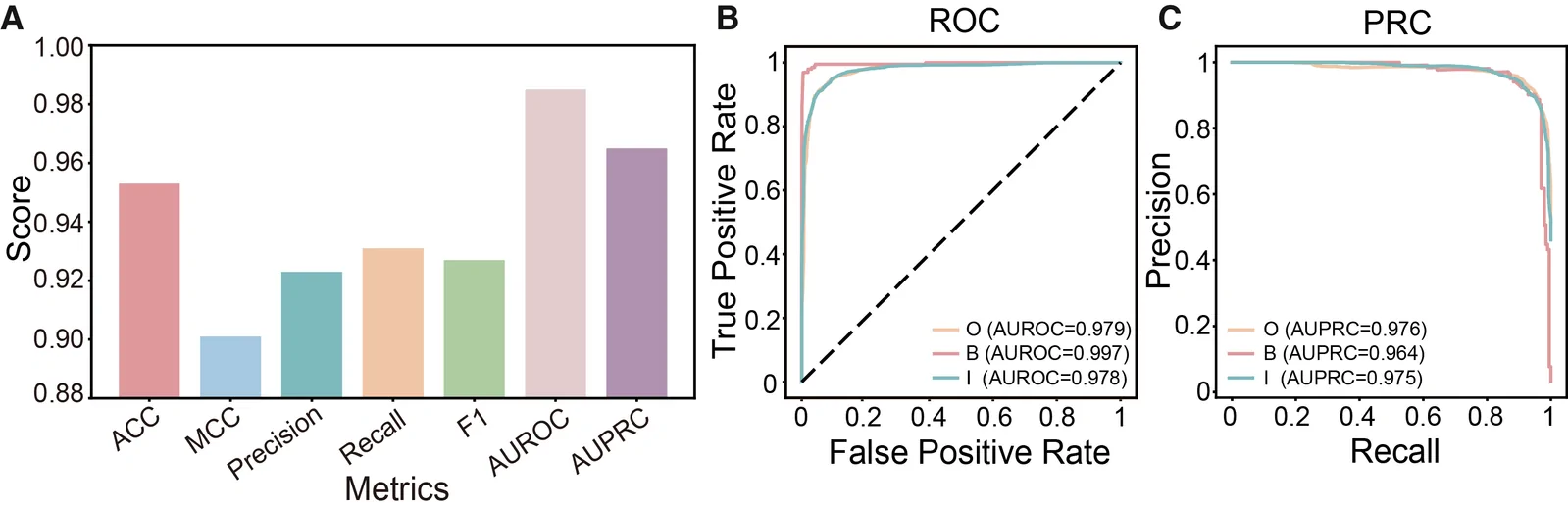
Performance of DeepNPA on the held-out test dataset (A) Summary bar plot showing all evaluation metrics. (B) ROC for each of the BIO tags. (C) PRC for each of the BIO tags.

The ROC curves (Figure 3B) demonstrated AUROC values of 0.979 (O), 0.997 (B), and 0.978 (I), reflecting excellent class separability. On the PRC (Figure 3C), DeepNPA achieved AUPRC values of 0.976 (O), 0.964 (B), and 0.975 (I), indicating consistently high precision across a wide range of recall values. This suggests that DeepNPA is capable of accurately detecting minority classes even when recall is high, a particularly important property in the context of imbalanced BIO tagging tasks. These results further indicate that the model maintains high precision while improving recall for minority classes.

Taken together, these results affirm that DeepNPA not only delivers superior classification accuracy but also maintains strong discriminative power and resilience to severe class imbalance, a common challenge in neuropeptide precursor cleavage site prediction.

### Contributions of pretraining and optimization strategies

To systematically quantify the contributions of architectural design and optimization strategies in DeepNPA, we conducted a systematic comparative study across six model variants using the default parameter settings, including general-purpose bidirectional encoder representations from transformers (BERT) and protein-specific ProtBert encoders, each combined with cross-entropy (CE), label smoothing (LS), or FL. Performance was evaluated using confusion matrices (Figures 4A–4F), classification metrics (Table 2), and probabilistic curves (Figures 4G and 4H).As shown in Figures 4A–4C, BERT-based variants exhibited limited discriminative capacity across the BIO tag space. Substantial bidirectional confusion between O and I classes was consistently observed, along with unstable identification of B (peptide boundary) residues. Specifically, BERT-CE (Figure 4A) showed extensive cross-class errors between O and I tags, indicating insufficient contextual representation for residue-level discrimination. BERT-LS (Figure 4B) slightly reduced misclassification within the O class but increased the misclassification of I residues as O and reduced recall for the B class, suggesting a bias toward the majority class induced by LS. BERT-FL (Figure 4C) improved B-class detection but did not resolve persistent cross-class confusion, with increased misclassification of O residues as B, indicating limited effectiveness in handling minority classes. To further evaluate the contribution of encoder architecture, we first compared BERT and ProtBert under the same CE optimization strategy. Contrary to the expectation that protein-specific pretraining alone would improve prediction performance, simply replacing the BERT encoder with ProtBert did not consistently enhance residue-level classification. Compared with BERT-CE (Figure 4A), ProtBert-CE (Figure 4D) still exhibited substantial confusion between the O and I classes, with increased O-to-I misclassification, indicating that protein-specific contextual representations alone were insufficient to improve cleavage site prediction under severe class imbalance.

**Figure 4:**
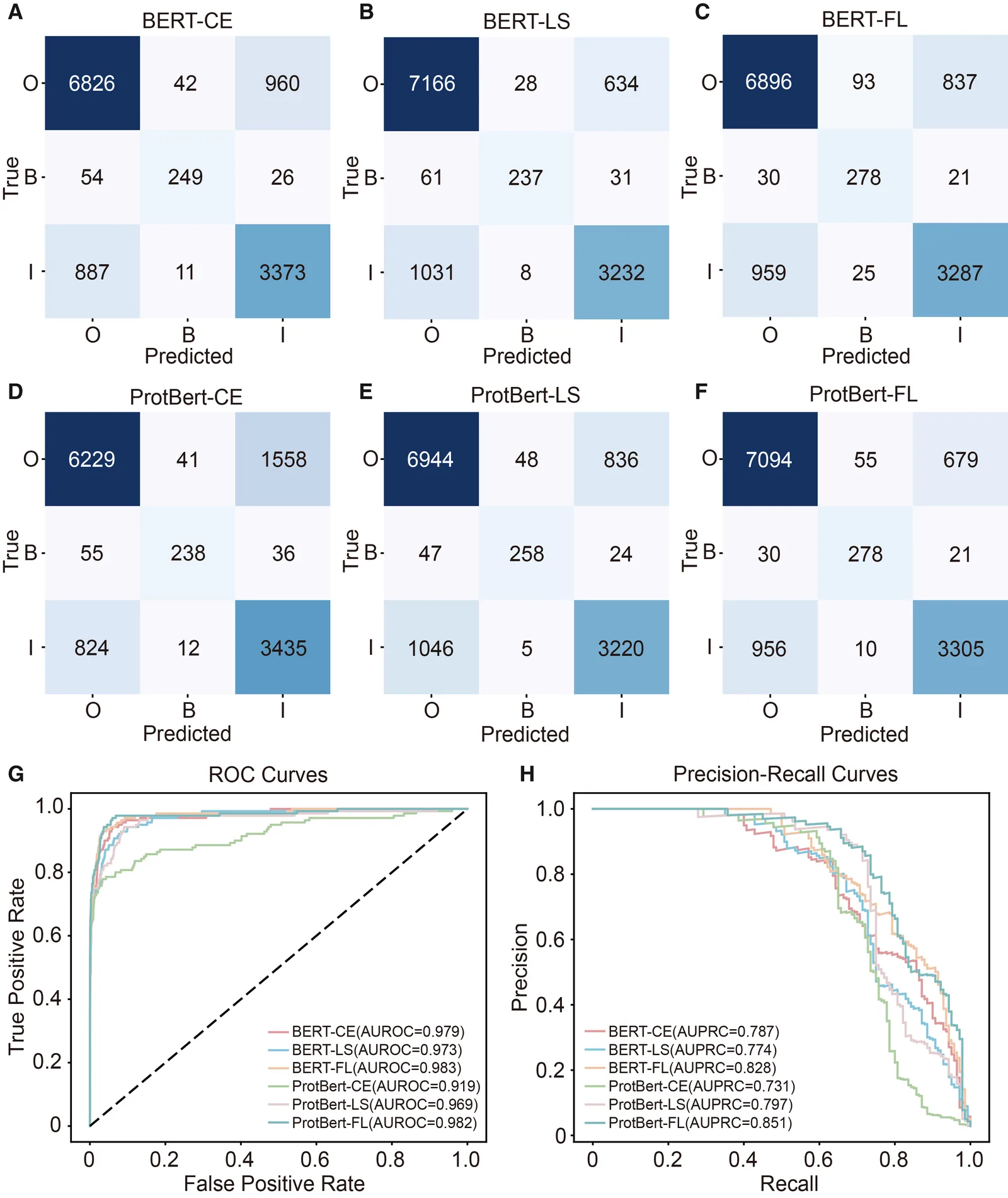
Ablation study comparing six model variants across confusion matrices, ROC curves, and precision–recall (PR) curves (A–C) Confusion matrices for BERT-based models using (A) cross-entropy, (B) label smoothing, and (C) focal loss. (D–F) Confusion matrices for ProtBert-based models using (D) cross-entropy, (E) label smoothing, and (F) focal loss. (G) ROC curves of the six models for class B. (H) PR curves for the six models for class B.

**Table 2:** Performance comparison of BERT and ProtBert-based model variants under different loss functions

Model	ACC	MCC	Precision	Recall	F1	AUROC	AUPRC
BERT_CE	0.910	0.831	0.867	0.837	0.848	0.979	0.787
BERT_LS	0.917	0.841	0.876	0.841	0.855	0.973	0.774
BERT_FL	0.918	0.844	0.877	0.857	0.865	0.983	0.828
ProtBert_CE	0.878	0.781	0.836	0.816	0.821	0.919	0.731
ProtBert_LS	0.914	0.837	0.874	0.839	0.852	0.969	0.797
ProtBert_FL	0.926	0.857	0.887	0.858	0.869	0.982	0.851

We next investigated whether optimization strategies could compensate for these limitations. ProtBert-LS (Figure 4E) further reduced O-to-I misclassification, improving majority-class precision while increasing I-to-O errors, reflecting a trade-off between precision and sensitivity. Notably, ProtBert-FL (Figure 4F) achieved the most balanced performance by minimizing cross-class errors while maintaining high precision for the dominant O class and stable recall for minority classes. The resulting confusion matrix exhibited the highest structural consistency, indicating improved robustness under class imbalance.

These observations are further supported by ROC and PRC analyses (Figures 4G and 4H). Consistent with the confusion matrix analysis, ProtBert-CE achieved lower AUROC (0.919) and AUPRC (0.731) than BERT-CE (AUROC = 0.979, AUPRC = 0.787), further indicating that protein-specific pretraining alone did not improve probabilistic discrimination. Following optimization with FL, ProtBert-FL achieved an AUROC of 0.982 and the highest AUPRC of 0.851, outperforming ProtBert-CE (AUROC = 0.919, AUPRC = 0.731) in the precision-recall space while maintaining comparable AUROC. This indicates a stronger ability to maintain precision at higher recall levels, which is critical for detecting minority classes (B and I) in imbalanced sequence labeling tasks. Although BERT-FL achieved a slightly higher AUROC (0.983), the superior AUPRC of ProtBert-FL suggests better practical performance for minority classes detection.

As summarized in Table 2, BERT-based models exhibited comparatively lower and less stable performance. The best-performing BERT variant (BERT-FL) achieved ACC of 0.918, F1 of 0.865, and Matthews correlation coefficient (MCC) of 0.844, with limited gains across different loss functions. For the ProtBert-based models, optimization strategy exerted an even greater influence on predictive performance. Although ProtBert-CE performed worse than BERT-CE, replacing cross-entropy with FL substantially improved all evaluation metrics. ProtBert-FL achieved the best overall performance, with ACC of 0.926, F1 of 0.869, and MCC of 0.857. Taken together, the best performance was achieved through the synergistic combination of the ProtBert encoder and FL, suggesting that protein-specific contextual representations become substantially more effective when coupled with an optimization strategy specifically designed to address severe class imbalance. This synergistic interaction, rather than either component alone, represents the principal performance advantage of DeepNPA.

### Hyperparameter sensitivity analysis

We conducted a comprehensive grid search to evaluate the influence of hyperparameter settings on model performance. The search space included three parameters: the maximum input sequence length (max_len) of ProtBert with values of 256 and 512, the batch size (bs) with values of 4, 8, 16, and 32, and the learning rate (lr) with values of 1e−5, 2e−5, 3e−5, 4e−5, and 5e−5. For FL, the hyperparameters *γ* (gamma) and *α*_*t*_ (class weights) were systematically explored across multiple configurations. Specifically, the candidate values for the class-weighting parameter *α*_*t*_ were,^6^^,^^8^^,^^10^ while the focusing parameter *γ* was evaluated over the range (1.0, 1.5, 2.0, and 2.5). Based on the aggregated ranking of multiple evaluation metrics, including ACC, F1-score, AUPRC, and AUROC, six top-performing configurations were selected for detailed analysis. The corresponding confusion matrices are shown in Figures 5A–5F.

**Figure 5:**
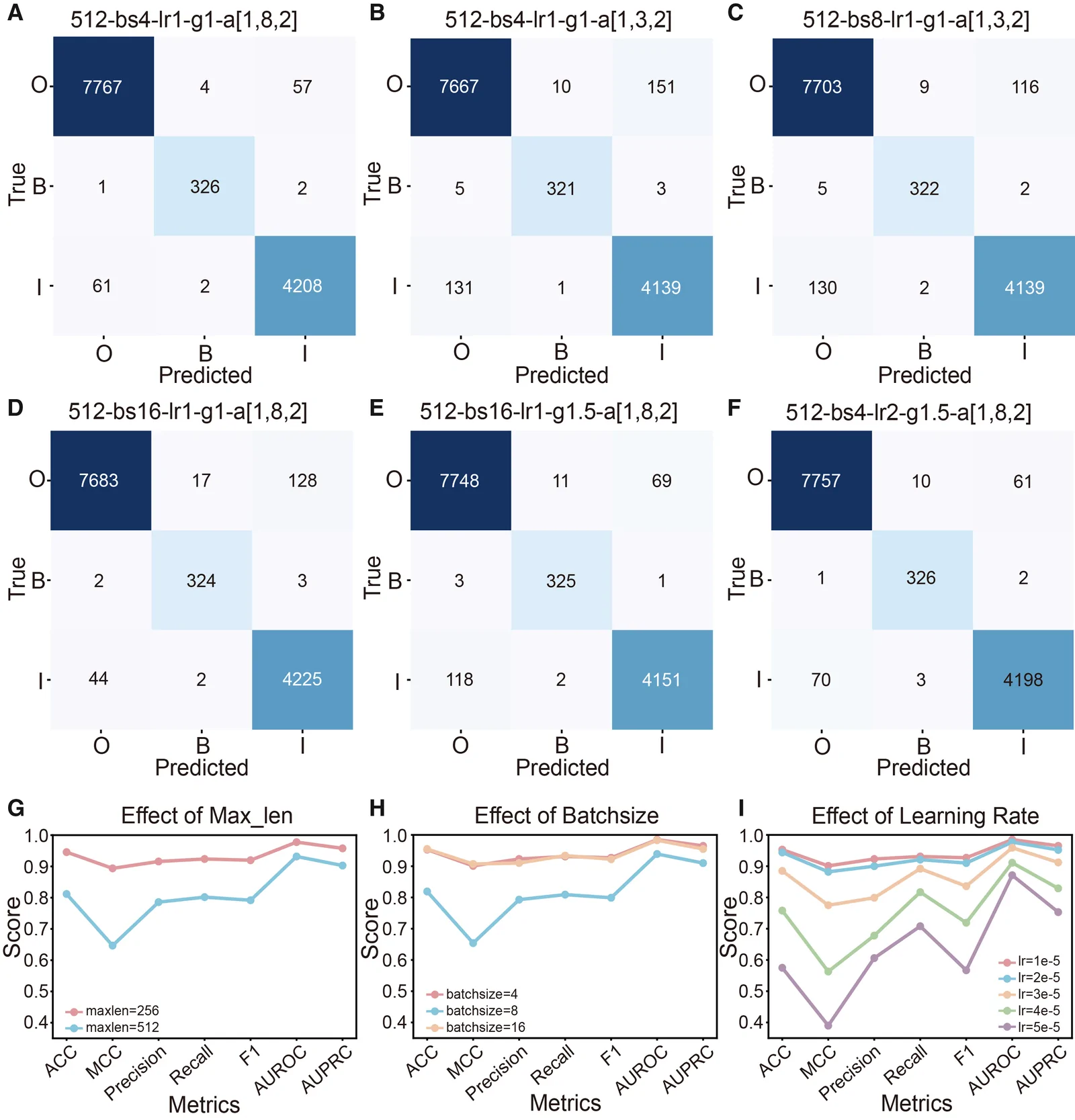
Performance comparison of DeepNPA under different hyperparameter configurations (A–F) Confusion matrices of the six top-ranked DeepNPA models trained under varying combinations of maximum input sequence length (max_len), batch size (bs), learning rate (lr), and focal loss parameters (*γ*, *α*_*t*_). (A) max_len = 512, bs = 4, lr = 1e−5, *γ* = 1, *α*_*t*_ = (1, 8, 2). (B) max_len = 512, bs = 4, lr = 1e−5, *γ* = 1, *α*_*t*_ = (1, 3, 2). (C) max_len = 512, bs = 8, lr = 1e−5, *γ* = 1, *α*_*t*_ = (1, 3, 2). (D) max_len = 512, bs = 16, lr = 2e−5, *γ* = 1, *α*_*t*_ = (1, 8, 2). (E) max_len = 512, bs = 16, lr = 1e−5, *γ* = 1.5, *α*_*t*_ = (1, 8, 2). (F) max_len = 512, bs = 4, lr = 2e−5, *γ* = 1.5, *α*_*t*_ = (1, 8, 2). (G–I) Quantitative impact of individual hyperparameters on model performance. (G) Comparison of models trained with max_len of 256 and 512 (bs = 4, lr = 1e−5, *γ* = 1, *α*_*t*_ = [1, 8, 2]). (H) Effect of batch size (4, 8, and 16) (max_len = 512, lr = 1e−5, *γ* = 1, *α*_*t*_ = [1, 8, 2]). (I) Impact of learning rate (1e−5, 2e−5, 4e−5, 5e−5, and 1e−6) (max_len = 512, bs = 4, *γ* = 1, *α*_*t*_ = [1, 8, 2]).

Among all configurations, the model using a maximum sequence length of 512, a batch size of 4, and a learning rate of 1e−5, with FL parameters *γ* = 1 and *α*_*t*_ = (1, 8, and 2), produced the most favorable results. This configuration achieved ACC of 0.953, MCC of 0.901, F1-score of 0.927, AUPRC of 0.965, and AUROC of 0.985, as shown in Table 3. As shown in Figure 5A, this model correctly classified 7,767 samples as tag O, 326 as tag B, and 4,208 as tag I. Only a small number of cross-class errors were observed. These extremely low misclassification rates reflect the model’s strong ability to discriminate between class boundaries and to accurately identify both major and minor classes.

**Table 3:** Top 6 models with optimal performance under different parameters

max_len	bs	lr	*γ*	*α*_*t*_[O, B, I]	ACC	MCC	Precision	Recall	F1	AUROC	AUPRC
512	16	1E−5	1	[1, 8, 2]	**0.955**	**0.907**	0.910	0.934	0.922	0.983	0.955
512	16	1E−5	1.5	[1, 8, 2]	0.954	0.904	0.916	**0.936**	0.925	0.980	0.955
**512**	**4**	**1E−5**	**1**	**[1, 8, 2]**	0.953	0.901	**0.923**	0.931	**0.927**	**0.985**	**0.965**
512	8	1E−5	1	[1, 3, 2]	0.949	0.894	0.912	0.918	0.915	0.981	0.951
512	4	1E−5	1	[1, 3, 2]	0.949	0.894	0.913	0.922	0.917	0.980	0.944
512	16	2E−5	1.5	[1, 8, 2]	0.948	0.891	0.905	0.932	0.918	0.980	0.954

Bold values in the parameter columns denote the optimal parameter combination, defined as the parameter set yielding the best overall performance. Bold values in the evaluation metric columns denote the highest value for each evaluation metric.

In contrast, other configurations led to significantly increased classification errors. The most prominent issue observed in suboptimal models was the misclassification between the O and I categories. For instance, the configuration using a maximum sequence length of 512, a batch size of 8, and a learning rate of 1e−5 (Figure 5B) resulted in 151 O-class samples being predicted as I and 131 I-class samples being predicted as O. A similar trend was evident in other settings such as the configuration in Figure 5D, which showed 128 O to I and 44 I to O misclassifications. These findings suggest that the ability to differentiate non-neuropeptide residues from internal neuropeptide regions is highly sensitive to training parameters.

To further analyze the individual effect of each hyperparameter, we performed one-variable perturbation experiments using the optimal model as the reference. In this procedure, one parameter was altered while keeping the others fixed. As shown in Figure 5G, models trained with a maximum sequence length of 512 consistently outperformed those using a length of 256 across most metrics (ACC, MCC, AUPRC, and AUROC), indicating that longer sequences capture more comprehensive contextual information for residue-level classification. The effect of batch size is illustrated in Figure 5H. A batch size of 8 or 16 provided comparable performance, with batch size 16 yielding slightly higher MCC and F1-score, while batch size 8 achieved marginally better AUPRC and AUROC. This suggests that moderate batch sizes stabilize gradient estimation without sacrificing fine-grained feature learning. Finally, the influence of learning rate is depicted in Figure 5I. The rates of 2e−5 and 1e−5 were found to be optimal, offering a balance between convergence stability and prediction accuracy. Conversely, a learning rate of 5e−5 resulted in the poorest performance across most metrics, likely due to excessive step sizes causing optimization instability and preventing fine convergence. These results demonstrate that careful selection of hyperparameters is essential for achieving optimal performance in cleavage site prediction.

### Comparative evaluation with state-of-the-art models

To comprehensively evaluate the predictive performance of DeepNPA, we conducted a comparative analysis against two representative methods for neuropeptide cleavage site prediction, NeuroCS and NeuroPred. Predictions for NeuroCS and NeuroPred were obtained using their publicly available online servers under default settings. For NeuroPred, the latest available model, “human amino acids plus properties neural net”, was selected for prediction.

As summarized in Table 4, DeepNPA outperformed both NeuroCS and NeuroPred across all evaluated metrics in the cleavage site identification task. Specifically, DeepNPA achieved a precision of 0.640, substantially higher than that of NeuroCS (0.350) and NeuroPred (0.557). This improvement indicates a markedly lower false-positive rate, suggesting that DeepNPA provides more reliable cleavage site predictions. In terms of recall, DeepNPA reached 0.723, exceeding NeuroCS (0.379) and NeuroPred (0.404). This result demonstrates an enhanced ability to identify true cleavage sites, which is critical for comprehensive neuropeptide precursor annotation and minimizing missed functional sites. The F1-score of DeepNPA was 0.681, outperforming NeuroCS (0.364) and NeuroPred (0.468), indicating a well-balanced performance between precision and recall. Furthermore, DeepNPA achieved higher values for MCC (0.666), AUROC (0.854), and AUPRC (0.478), reflecting improved overall classification performance and robustness under class-imbalanced conditions.

**Table 4:** Comparative performance of NeuroCS, NeuroPred, and DeepNPA

Model	ACC	MCC	Precision	Recall	F1	AUROC	AUPRC
NeuroCS	0.939	0.333	0.350	0.379	0.364	0.673	0.161
NeuroPred	0.952	0.450	0.557	0.404	0.468	0.693	0.256
DeepNPA	0.969	0.666	0.640	0.723	0.681	0.854	0.478

Overall, the consistent performance gains observed across all metrics under a strictly non-overlapping evaluation setting suggest that the predictive advantage of DeepNPA is not attributable to dataset redundancy, but rather to its improved modeling capacity.

### Interpretation of learned representations using t-SNE

To investigate the internal feature-learning behavior of DeepNPA, we applied t-distributed stochastic neighbor embedding (t-SNE) to visualize hidden representations extracted from multiple layers of the ProtBert encoder (Figure 6). Four representative layers were selected for analysis, including the input embedding layer (layer 0), an early intermediate layer (layer 4), a deep encoder layer (layer 12), and the classifier output layer. Attention masks were applied to remove padding tokens, ensuring that only valid residue representations contributed to the visualization. Emission scores from the classifier were also included to reflect the final decision space.

**Figure 6:**
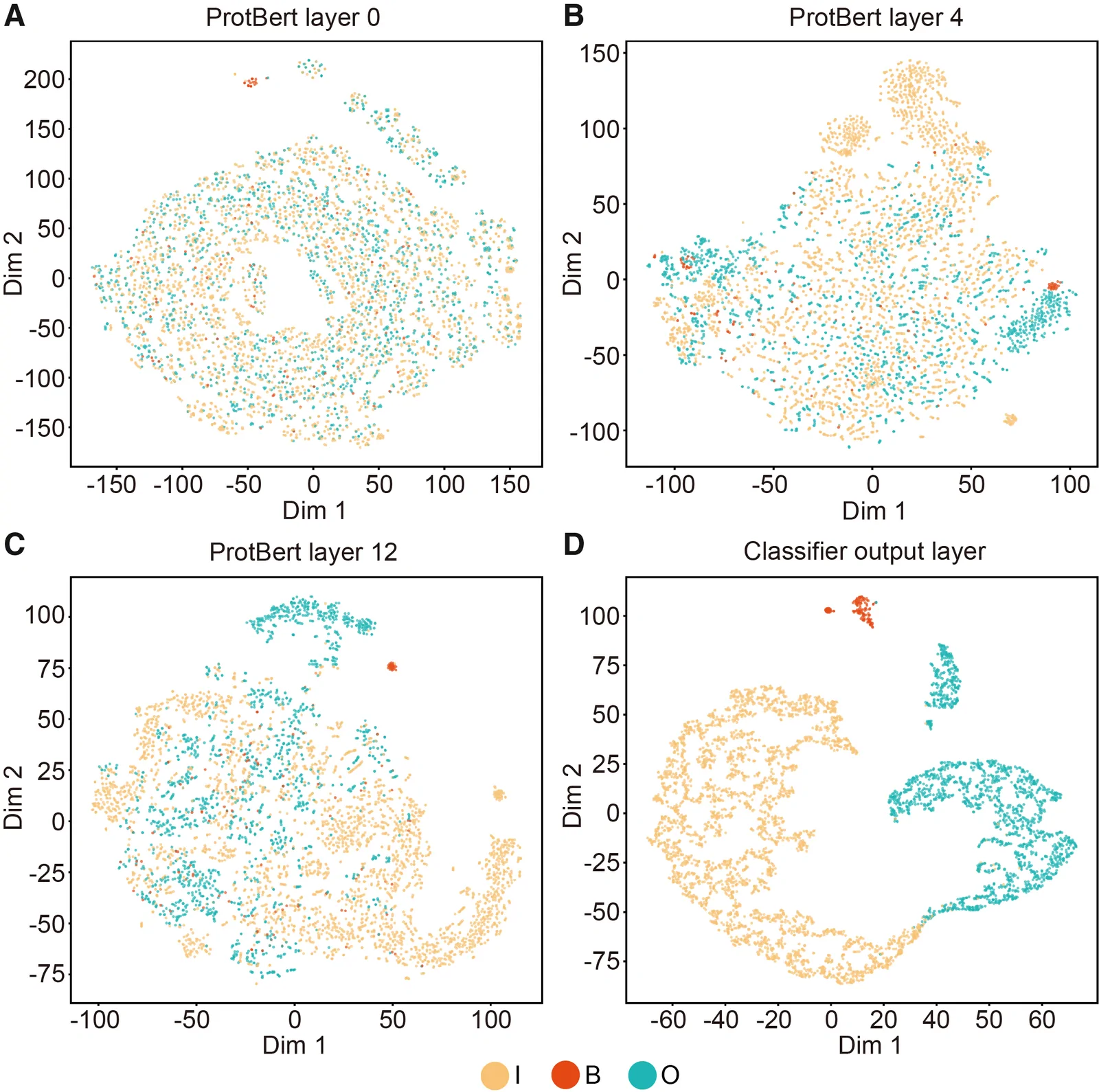
t-SNE visualization of hidden representations across ProtBert layers (A) Layer 0 (input embedding layer). (B) Layer 4 (intermediate layer). (C) Layer 12 (deep encoder layer). (D) Classifier output layer.

The visualizations reveal a clear hierarchical progression in feature organization. Layer 0 (input embedding layer) shows nearly complete overlap among B, I, and O tags, indicating that token representations at this stage contain minimal category-specific information and primarily reflect raw sequence encoding (Figure 6A). By layer 4 (early intermediate layer), weak clustering tendencies begin to emerge. The B and I classes show partial grouping, although considerable overlap remains among all three tags (Figure 6B). This suggests the early extraction of coarse category-relevant features, yet with limited discriminative capability. In layer 12 (deep encoder layer), label-specific structures become substantially more defined (Figure 6C). The B class forms compact and clearly separated clusters, while the O and I classes show much stronger internal cohesion despite some remaining overlap. This indicates that deeper layers successfully embed more abstract and discriminative semantic information derived from long-range sequence context and multi-head attention mechanisms. At the classifier output layer, class boundaries are most clearly established. The B class remains tightly clustered and well isolated, O forms distinct groupings, and only a small subset of I tags overlaps with O tags. These overlapping samples correspond to residues with lower prediction confidence, reflecting intrinsic ambiguity in local sequence environments (Figure 6D).

Overall, the t-SNE results demonstrate that ProtBert exhibits a progressive enhancement of discriminative power with increasing network depth. The model transitions from raw sequence encoding to refined semantic abstraction and ultimately to decision-level feature separation. Notably, the B class achieves early and strong representation separation, whereas the I and O classes display finer-grained distinctions and inherently more challenging boundaries.

### External validation of DeepNPA

To further evaluate the generalization capability of DeepNPA, we constructed an external validation dataset using experimentally validated neuropeptide data reported in a recent mass spectrometry-based study of the neuropeptidomes of Stichopus cf. horrens and Holothuria scabra.^16^ This dataset provides a realistic benchmark for assessing model performance on previously unseen species. A total of 16 neuropeptide precursor sequences were collected, covering multiple families, including THR-like, calcitonin-type, and cholecystokinin-type precursors. Residue-level annotations were generated based on experimentally validated mature peptide regions using the BIO tagging scheme.

Compared with existing methods, DeepNPA demonstrated consistently better performance than existing methods, particularly in identifying minority class residues. Specifically, DeepNPA achieved an MCC of 0.394 and an F1-score of 0.428 (Table 5), markedly outperforming NeuroCS (MCC: 0.056, F1: 0.033) and NeuroPred (MCC: 0.083, F1: 0.076). These results indicate that DeepNPA is more effective at identifying proteolytic cleavage sites and recovering neuropeptide regions within highly imbalanced sequences.

**Table 5:** Performance of DeepNPA and existing methods on the external validation dataset

Model	ACC	MCC	Precision	Recall	F1	AUROC	AUPRC
DeepNPA	0.920	0.394	0.522	0.362	0.428	0.666	0.242
NeuroCS	0.916	0.056	0.308	0.017	0.033	0.507	0.086
NeuroPred	0.912	0.083	0.286	0.044	0.076	0.517	0.091

Since the external validation dataset was derived from echinoderms, which belong to a distinct phylum from the species used in model training, this evaluation represents a cross-phylum validation scenario. Compared to its performance on the independent benchmark dataset, a decrease in performance is observed on this dataset, which can be reasonably attributed to the cross-phylum nature of the validation species. As evolutionary divergence increases, neuropeptide precursors exhibit reduced sequence conservation, leading to differences in sequence context and precursor organization that may affect cleavage site recognition. Despite these challenges, DeepNPA retains the capacity to identify neuropeptide regions and achieves moderate performance (AUROC = 0.666), indicating a certain level of robustness under cross-phylum distributional shifts.

Visual inspection of representative predictions further supports these quantitative results (Figure 7). DeepNPA simultaneously predicts cleavage sites and mature neuropeptide regions, whereas comparison methods are restricted to cleavage site prediction. Across multiple precursor families, DeepNPA’s predictions show high concordance with experimentally validated tags, accurately capturing both cleavage positions and contiguous peptide segments. This is particularly evident in complex precursors containing multiple segments, where DeepNPA consistently recovers regions with precise boundaries. In contrast, NeuroPred and NeuroCS identify only a limited subset of cleavage sites and often produce fragmented or incomplete outputs, failing to reconstruct the full peptide architecture. The improved performance of DeepNPA is particularly evident in complex precursors containing multiple peptide segments, where accurate boundary delineation is required.

**Figure 7:**
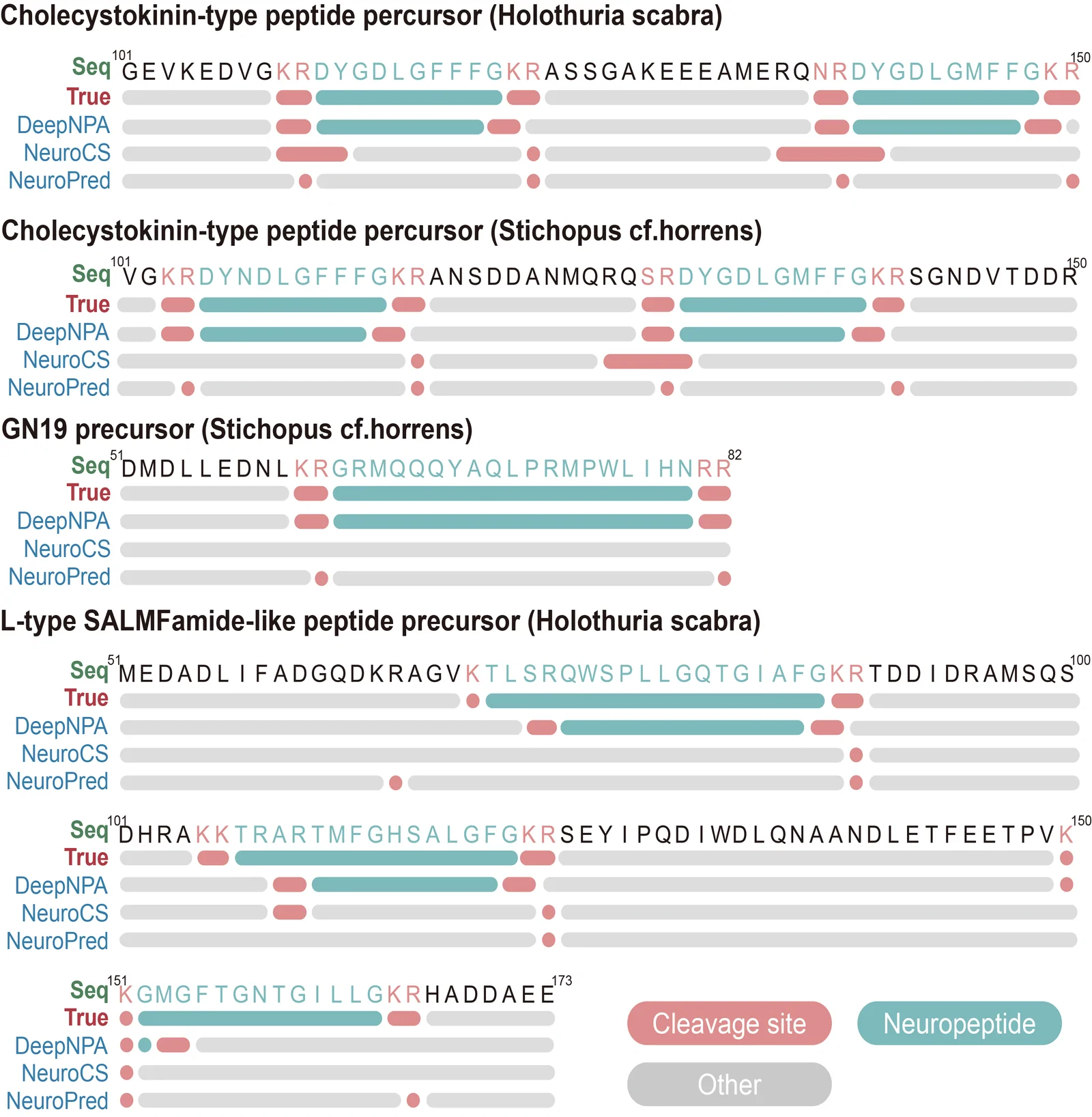
Visualization of neuropeptide precursor prediction in external validation dataset The amino acid sequences are shown at the top of each block, where the superscript numbers denote the amino acid positions in the precursor sequence. Red represents the cleavage sites; green represents the neuropeptide sequences; and gray represents the non-neuropeptide regions of the precursor sequence.

To further assess the biological plausibility of the predictions, we analyzed the motif distribution of predicted cleavage sites. The results showed that most predicted sites were located at canonical dibasic motifs, particularly KR (Lys–Arg) and RR (Arg–Arg),^17^^,^^18^ consistent with known recognition preferences of proprotein convertases (Figure 7). Moreover, motifs such as GK (Gly–Lys) were observed in representative sequences.^19^ This pattern is consistent with known neuropeptide processing signals, where glycine residues often serve as amide donors for C-terminal amidation, and cleavage frequently occurs near basic residues. These findings further indicate that the model captures biologically relevant processing signals.

### Web server implementation for DeepNPA

To facilitate practical application, we developed a publicly accessible web server for DeepNPA, available at https://i-health.info/DeepNPA/. The platform enables users to perform neuropeptide precursor cleavage site prediction using the pretrained model without requiring local computational resources or programming expertise.

The server supports two input modes: users can either paste protein sequences directly into a submission panel or upload FASTA-format files containing one or multiple sequences. Upon submission, the backend system automatically processes the input and performs inference using the pretrained DeepNPA model.

For sequences exceeding the model’s maximum input length (512 amino acids), a sliding-window strategy is applied. Specifically, long sequences are divided into overlapping fragments, predictions are generated for each fragment, and the outputs are subsequently merged to produce residue-level annotations. This design enables the model to handle long protein sequences while preserving contextual information near fragment boundaries. To ensure stable performance in the public server environment, the maximum supported sequence length is currently set to 1,500 amino acids per sequence. Inputs exceeding this limit are rejected with an automatic notification. The server was evaluated on a system equipped with an Intel Xeon Silver 4215R CPU (16 cores) and 125 GB RAM. Under typical conditions, the average processing time is approximately 2–5 s per sequence, depending on sequence length and server load.

Prediction results are presented at the residue level using the BIO tagging scheme, where B indicates the start of a neuropeptide segment, I denotes internal residues, and O represents non-neuropeptide regions. For improved interpretability, the output is visualized using color coding (red for B, blue for I, and gray for O), allowing users to quickly identify predicted cleavage sites and peptide regions (Figure 8).

**Figure 8:**
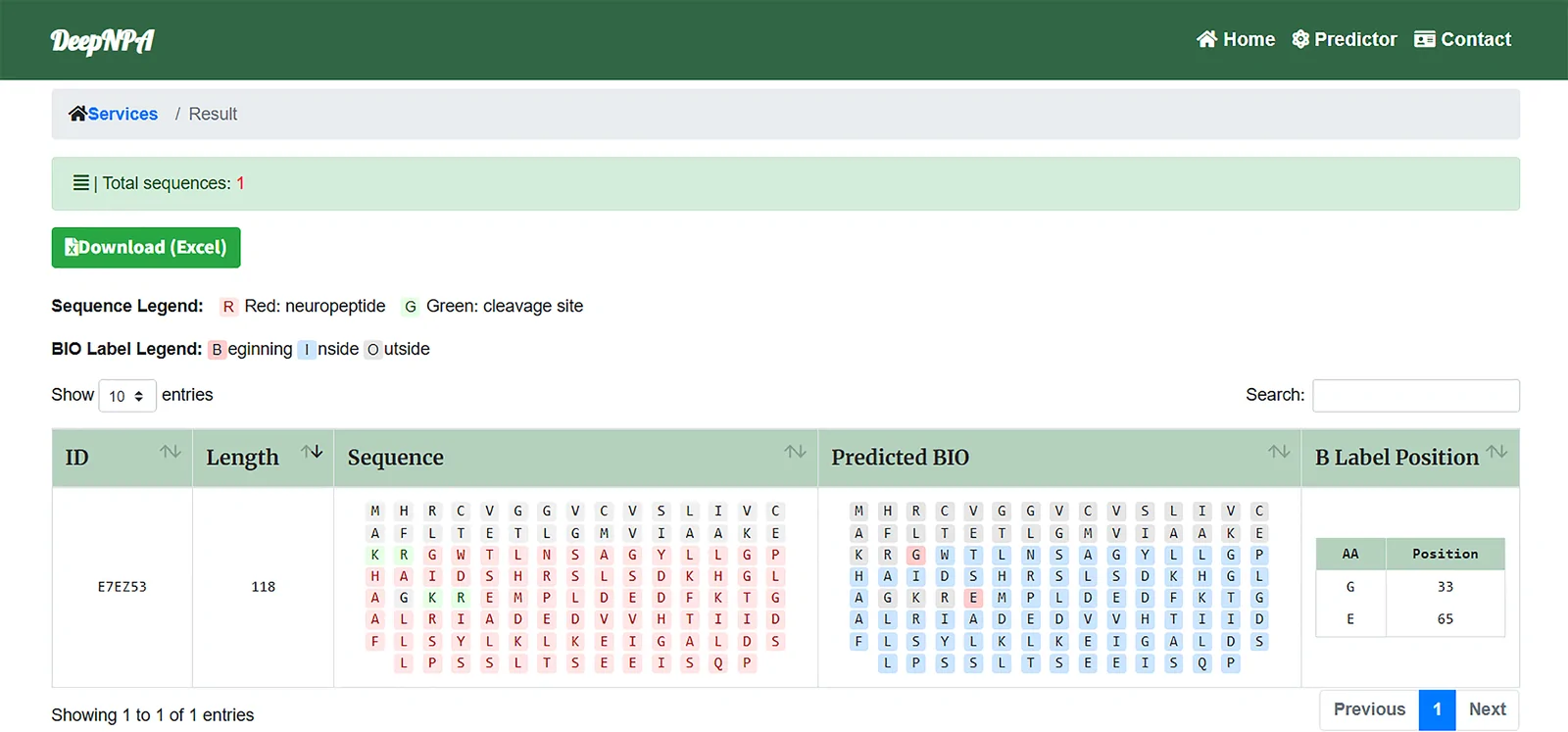
Visualization of DeepNPA prediction results from the online web server

Overall, the web server provides a user-friendly interface for applying DeepNPA to neuropeptide precursor analysis and supports downstream experimental investigation.

## Discussion

This study addresses the task of neuropeptide precursor cleavage site prediction by integrating standardized dataset construction, model development, and practical deployment. The overall framework provides a systematic approach for evaluating and applying sequence-based prediction methods in neuropeptide informatics. We constructed a curated dataset of neuropeptide precursors with unified BIO-format annotations and established a multi-model, multi-metric evaluation protocol. This benchmark facilitates reproducible comparison across different methods. Based on this dataset, DeepNPA was developed as a sequence labeling framework combining ProtBert representations with FL to address class imbalance. Under this setting, DeepNPA achieves improved performance compared to existing tools, including NeuroCS and NeuroPred, with an ACC of 0.920 on the independent benchmark dataset. The performance improvements observed for DeepNPA are likely attributable to the complementary strengths of contextual sequence representation and imbalance-aware optimization. ProtBert captures long range contextual dependencies that may contribute to cleavage site recognition, Meanwhile, FL reduces the dominance of abundant non-cleavage residues during optimization, allowing the model to focus more effectively on difficult and biologically relevant cleavage positions.

Hyperparameter analysis suggests that model performance is sensitive to training configurations but remains relatively stable within a reasonable range. A maximum sequence length of 512 consistently yields strong performance, indicating the importance of contextual information for cleavage site identification. Furthermore, the use of FL with tuned class weights enhances the detection of rare cleavage residues, supporting its utility in highly imbalanced settings.

In comparative evaluations, DeepNPA demonstrates consistent improvements over existing methods, achieving higher precision and recall simultaneously. This balanced performance indicates reduced false positives while maintaining sensitivity, which is important for reliable neuropeptide annotation.

External validation on echinoderm datasets provides additional insight into model generalization. Although performance decreases relative to the in-domain test set (e.g., AUROC = 0.666, AUPRC = 0.242), DeepNPA maintains a clear advantage over baseline methods, particularly in MCC and F1-score. The reduced performance observed during external validation is likely attributable to several factors, including evolutionary divergence between training and testing species, differences in sequence composition, and species-specific processing mechanisms. Nevertheless, the ability of DeepNPA to maintain competitive performance on phylogenetically distant organisms suggests that the learned representations possess meaningful biological generalizability rather than merely memorizing training examples. These results suggest that the model captures sequence patterns that are at least partially transferable across species despite phylogenetic differences. To support practical use, a web server was implemented to enable cleavage site prediction without requiring specialized computational resources. This platform provides a convenient interface for applying DeepNPA in neuropeptide-related studies.

Recent advances in protein foundation models and structure-aware deep learning have increasingly enabled the integration of sequence and structural information for protein function prediction. Incorporating structural features, such as predicted three-dimensional structures, solvent accessibility, residue flexibility, residue contact maps, and local conformational dynamics, may provide complementary information that is not readily captured by linear amino acid sequences alone. In future work, we will consider extending DeepNPA by integrating protein language model representations with such structure-aware features to better characterize the sequence-structure determinants underlying neuropeptide precursor processing. In parallel, continued improvements in experimental resources will be essential for developing more comprehensive and generalizable prediction models. As large-scale peptidomics and proteomics datasets become available across a broader range of phylogenetically diverse organisms, they will provide increasingly valuable resources for model training and independent validation. Incorporating experimentally supported biological context, including species- and tissue-specific processing information, may further help characterize processing patterns that cannot be fully inferred from sequence or structural information alone. Together, the integration of sequence information, structural features, biological context, and experimentally supported knowledge may provide a promising framework for advancing residue-level annotation of neuropeptide precursors. Beyond improving cleavage site prediction, such integrative approaches may also advance our understanding of the molecular determinants of neuropeptide maturation and facilitate the discovery of previously uncharacterized bioactive neuropeptides across diverse metazoan species.

### Limitations of the study

Despite the observed performance improvements, several limitations remain. First, although manually reviewed UniProtKB/Swiss-Prot annotations provide a high-quality reference for supervised learning, some mature neuropeptide annotations may not yet have direct experimental confirmation by peptidomics or mass spectrometry. In addition, the current external evaluation is based on a relatively small number of experimentally validated sequences, which restricts comprehensive assessment across broader phylogenetic ranges. Second, extreme class imbalance remains a fundamental challenge. In the external validation dataset, cleavage sites account for approximately 2% of residues, which negatively impacts recall and F1-score. Although FL mitigates this issue to some extent, further improvements are needed for detecting weak or non-canonical cleavage signals. Third, neuropeptide precursor processing is inherently influenced by biological context beyond amino acid sequence alone. Although precursor sequence provides the primary information for cleavage site recognition, the generation of mature neuropeptides is further regulated by lineage specific differences in prohormone processing enzymes, tissue-specific expression of prohormone convertases, developmental stage, physiological status, subcellular localization, and other cellular contexts. Consequently, DeepNPA is designed as a sequence-based predictor that learns statistical mappings between precursor sequences and experimentally curated residue annotations, enabling the identification of conserved sequence features associated with neuropeptide processing. However, it cannot explicitly model context-dependent processing events determined by species, tissue, or physiological specific factors. This biological complexity likely represents an intrinsic upper bound for purely sequence-based prediction models.

The limitations identified in this study suggest several directions for further development. While DeepNPA was designed as a sequence-based framework, incorporating complementary biological information may further improve its ability to model context-dependent neuropeptide processing. Phylogenetic relationships, tissue-specific expression patterns, developmental stage, and the distribution of prohormone convertases and other processing enzymes all influence precursor maturation and could provide valuable context beyond primary sequence alone.

## Resource availability

### Lead contact

Requests for further information and resources should be directed to and will be fulfilled by the lead contact, Juan-Juan Kang (kangjuanjuan@cdutcm.edu.cn).

### Materials availability

This study did not generate new unique reagents.

### Data and code availability

• All datasets used for model training, validation, and testing in this study, including both raw sequence data and processed datasets, have been deposited at GitHub: https://github.com/LUMEN-123/DeepNPA and archived at Zenodo: https://doi.org/10.5281/zenodo.22206809, and are publicly available as of the date of publication.
• All original code for DeepNPA, including model implementation, training scripts, and evaluation pipelines, has been deposited at GitHub: https://github.com/LUMEN-123/DeepNPA and archived at Zenodo: https://doi.org/10.5281/zenodo.22206809, and is publicly available as of the date of publication.
• All other data reported in the manuscript will be shared by the lead contact upon request.

## Acknowledgments

This work was supported by 10.13039/501100001809National Natural Science Foundation of China (no. 62302066 and 62501110), and 10.13039/501100002858China Postdoctoral Science Foundation (grant no. 2020TQ0138).

## Author contributions

Conceptualization, J.-J.K. and X.-Y.L.; methodology, X.-Y.L. and J.-J.K.; investigation, X.-Y.L., Y.Y., and L.-P.L.; writing – original draft, X.-Y.L. and J.-J.K.; writing – review and editing, J.-J.K and Q.T.; funding acquisition, J.-J.K.; supervision, J.-J.K., Q.T., and M.-Z.D.

## Declaration of interests

The authors declare no competing interests.

## Declaration of generative AI and AI-assisted technologies in the writing process

During the preparation of this work, the authors used DeepSeek in order to correct spelling and grammar errors and improve language fluency. After using this tool/service, the author(s) reviewed and edited the content as needed and takes full responsibility for the content of the publication.

## STAR★Methods

### Key resources table

**Table:** 

REAGENT or RESOURCE	SOURCE	IDENTIFIER
**Deposited data**		

Raw sequence information	UniProt et al.^14^	https://www.uniprot.org/
External validation	Resoles et al.^16^	https://www.nature.com/articles/s41598-025-85696-7
Dataset in this paper	GitHub	GitHub: https://github.com/LUMEN-123/DeepNPA
Dataset in this paper	Zenodo	Zenodo: https://doi.org/10.5281/zenodo.22206809

**Software and algorithms**		

ProtBert	Elnaggar et al.^12^	https://huggingface.co/Rostlab/prot_bert
CD-HIT (v4.8.1)	Fu et al.^20^	https://weizhongli-lab.org/cd-hit/
Python (v3.11)	Python Software Foundation	https://www.python.org/
PyTorch (v2.9.0)	Paszke et al.^21^	https://pytorch.org/
Transformers (v4.57.1)	Wolf et al.^22^	https://github.com/huggingface/transformers
Scikit-learn (v1.7.2)	Pedregosa et al.^23^	https://scikit-learn.org
NumPy (v2.3.3)	Harris et al.^24^	https://numpy.org
Matplotlib (v3.8.4)	Hunter, J.D.et al.^25^	https://matplotlib.org
NeuroPred	Southey et al.^10^	Model: Human amino acids plus properties Neural Net; https://neuroproteomics.scs.illinois.edu/neuropred.htm
NeuroCS	Wang et al.^8^	https://i.uestc.edu.cn/NeuroCS/dist/index.html?file=#/
Code in this paper	GitHub	GitHub: https://github.com/LUMEN-123/DeepNPA
Code in this paper	Zenodo	Zenodo: https://doi.org/10.5281/zenodo.22206809

### Method details

#### Data sources and preprocessing

We collected manually reviewed neuropeptide precursor sequences primarily from the UniProtKB/Swiss-Prot database. To construct a high-quality dataset, entries annotated with the keyword *Neuropeptide* (KW-0527) were retrieved, while those labeled with *Receptor* (KW-0675) were excluded to avoid contamination with receptor sequences. Reviewed (Swiss-Prot) records were included, whereas automatically annotated UniProtKB/TrEMBL entries were excluded. Residue-level mature neuropeptide annotations were obtained directly from the PTM/Processing section of the UniProtKB/Swiss-Prot entries, where the curated peptide feature positions were used as the reference mature neuropeptide boundaries. These residue positions were subsequently converted into BIO tags for model training. In total, we obtained 1,500 neuropeptide precursor sequences from 273 species across 7 phyla (*Arthropoda, Chordata, Nematoda, Mollusca, Annelida, Platyhelminthes* and *Cnidaria*), including key model organisms such as *Caenorhabditis elegans*, *Drosophila melanogaster*, *Homo sapiens*, *Mus musculus*, and *Rattus norvegicus*. According to the *Protein Existence* annotations in UniProt, 1,371 proteins (91.4%) were supported by experimental evidence at the protein level, 79 proteins (5.3%) by experimental evidence at the transcript level, and 50 proteins (3.3%) were inferred by homology. Thus, the dataset was predominantly composed of proteins with direct experimental support, while a small proportion of entries were supported by transcript-level evidence or high-confidence homology-based inference according to the UniProt evidence classification system. For data preprocessing, two core steps were implemented to standardize the dataset. First, the classical BIO (Begin-Inside-Other) tagging scheme was adopted to unify sequence annotations, where tag B denotes the first residue of a mature neuropeptide segment, I represents internal residues within a neuropeptide, and O corresponds to non-neuropeptide regions of the precursor. Second, CD-HIT^20^ was applied at a sequence identity threshold of 0.9 to eliminate highly similar sequences and reduce the risk of overfitting, resulting in 685 high-quality non-redundant sequences retained for subsequent model development (https://github.com/LUMEN-123/DeepNPA). The dataset was randomly divided into training, validation, and held-out test datasets at a ratio of 7:1:2 (Table 1).

To enable fair comparison with existing prediction tools, an independent benchmark dataset was constructed from the held-out test dataset. Sequences overlapping with the training datasets used by NeuroPred and NeuroCS were removed to avoid potential data leakage. Subsequently, CD-HIT was applied with a sequence identity threshold of 0.4 to further reduce redundancy. After filtering, 50 neuropeptide precursor sequences were retained for independent benchmark evaluation. These sequences comprise 30 species spanning 6 phyla (Arthropoda, Chordata, Cnidaria, Mollusca, Nematoda, Platyhelminthes), as summarized in Table S1.

In addition, to assess the external validation generalization of DeepNPA, we constructed an external validation dataset based on a recent neuropeptidomics study. In that study, neuropeptides were identified using mass spectrometry-based approaches, providing experimentally validated annotations for precursor proteins and their corresponding mature peptide regions. Based on these validated data, we collected 16 neuropeptide precursor sequences from *Stichopus* cf. *horrens* and *Holothuria scabra*.^16^ This dataset was subsequently used to evaluate model performance on previously unseen species and to assess its external validation generalization ability.

#### ProtBert encoder

ProtBert is a protein-specific language model derived from the BERT architecture and pretrained on large-scale protein sequence databases, including Uniref. 100 and the Big Fantastic Database (BFD).^12^ Compared with general-domain BERT models, ProtBert increases the encoder depth to 30 Transformer layers, each including a multi-head self-attention sublayer with 12 attention heads and a position-wise feedforward network. Residual connections and layer normalization are applied throughout the network to facilitate stable optimization. Through fully bidirectional self-attention, each residue representation can attend to all other positions in the sequence, enabling effective modeling of long-range dependencies and contextual interactions.

The self-attention operation is defined as:

(Equation 1) $$Attention\left(Q,K,V\right)=softmax\left(\frac{QK^{T}}{\sqrt{d_{k}}}\right)V$$

where *Q*, *K* and *V* denote the query, key, and value matrices, respectively, and *d*_*k*_ represents the dimensionality of the key vectors.

Through large-scale self-supervised pretraining, ProtBert learns rich residue-level representations that implicitly encode biochemical and structural properties of protein sequences. These representations provide a strong foundation for downstream sequence annotation tasks. In DeepNPA, ProtBert is employed as a fixed architecture backbone and fine-tuned end-to-end for neuropeptide precursor cleavage site identification.

#### Focal loss for imbalanced BIO tagging

Cleavage site prediction is inherently affected by severe class imbalance, as cleavage-related residues constitute only a small fraction of amino acids in neuropeptide precursor sequences. Under the BIO tagging scheme, the O tag dominates the dataset, whereas the B and I tags appear infrequently. When optimized using standard Cross-Entropy loss, models tend to bias toward the majority class, resulting in suboptimal detection of cleavage sites.

To address this issue, DeepNPA adopts FL^13^ as the training objective. FL dynamically down-weights the contribution of easily classified samples and emphasizes hard examples, thereby improving learning for minority classes. The loss function is defined as:

(Equation 2) $$FocalLoss\left(p_{t}\right)=-\alpha_{t}{\left(1-p_{t}\right)}^{\gamma }\;\log\left(p_{t}\right)$$

Here, *p*_*t*_ denotes the predicted probability of the true class. The weighting factor *α*_*t*_ denotes the class-specific weight and is set to 1, 8, and 2 for the O, B, and I classes, respectively, to increase the contribution of the minority B and I classes. The focusing parameter *γ* is set to 1.0, following the default value recommended in the original FL formulation, to down-weight the contribution of easily classified samples.

#### Sliding window segmentation

Protein sequences exceeding the maximum model input length (512 amino acids) were processed using a sliding-window segmentation strategy. Specifically, each sequence was divided into overlapping fragments of fixed length (512 residues), with an overlap of 64 residues between adjacent windows.

This overlapping design preserves local contextual information near fragment boundaries and mitigates potential prediction errors introduced by sequence truncation. In particular, it ensures that residues located near window edges are evaluated with sufficient surrounding context, thereby improving the consistency of residue-level predictions across segments.

#### Model performance evaluation metrics

Model performance was evaluated using Accuracy (ACC), Precision, Recall, F1-score, and Matthews Correlation Coefficient (MCC).^26^^,^^27^^,^^28^ The definitions of these metrics are given below.

Let $C\in R^{K\times K}$ denote the confusion matrix, where *C*_[*i*][*j*]_ represents the number of residues whose true class is *i* and predicted class is *j*, and *K* is the total number of classes.

Accuracy measures the proportion of correctly classified residues:

(Equation 3) $$Accuracy=\frac{\sum_{i=0}^{K-1}C_{\left[k\right]\left[k\right]}}{\sum_{i=0}^{K-1}\sum_{j=0}^{K-1}C_{\left[i\right]\left[j\right]}}$$

The numerator represents the total number of correctly classified residues (i.e., the sum of diagonal elements in the confusion matrix), while the denominator denotes the total number of residues.

Precision and Recall are computed using macro-averaging:

(Equation 4) $$Precision=\frac{1}{K}\sum_{i=0}^{k-1}\frac{C_{\left[k\right]\left[k\right]}}{\sum_{j=0}^{N-1}C_{\left[i\right]\left[k\right]}}$$

(Equation 5) $$Recall=\frac{1}{N}\sum_{i=0}^{K-1}\frac{C_{\left[k\right]\left[k\right]}}{\sum_{j=0}^{N-1}C_{\left[k\right]\left[j\right]}}$$

where *C*_[*k*][*k*]_ denotes the number of correctly predicted residues for class *i*, and *C*_[*i*][*k*]_ denotes the residues predicted as class *k*, *C*_[*k*][*j*]_ denotes the total number of residues that truly belong to class *k*. The off-diagonal elements *C*_[*i*][*j*]_(*i*≠*j*) correspond to misclassified residues.

F1-score is defined as the harmonic mean of Precision and Recall for each class and then averaged across classes:

(Equation 6) $$F1\;Score=\frac{1}{K}\sum_{i=0}^{K-1}\frac{2\cdot Precision_{k}\times Recall_{k}}{Precision_{k}+Recall_{k}}$$

This metric ensures equal contribution from each class and is particularly suitable for imbalanced classification tasks.

To provide a more comprehensive evaluation under class imbalance, we employed the Matthews correlation coefficient (MCC), generalized for multi-class classification:

(Equation 7) $$MCC=\frac{\sum_{k,l,m=1}^{N}C_{\left[k\right]\left[k\right]}C_{\left[m\right]\left[l\right]}-C_{\left[l\right]\left[k\right]}C_{\left[k\right]\left[m\right]}}{\sqrt{\sum_{k=1}^{N}\left[\left(\sum_{l=1}^{N}C_{\left[l\right]\left[k\right]}\right)\left(\sum_{f,g=1f\neq 1}^{N}C_{\left[g\right]\left[f\right]}\right)\right]}\sqrt{\sum_{k=1}^{N}\left[\left(\sum_{l=1}^{N}C_{\left[k\right]\left[l\right]}\right)\left(\sum_{f,g=1f\neq 1}^{N}C_{\left[f\right]\left[g\right]}\right)\right]}}$$

Where, *C* is the confusion matrix, *N* is the total number of classes, *k*,*l*,*m*,*f* and *g* are traversal indices covering all tag categories. The numerator computes the covariance between ground truth and predicted tag distributions, where *C*_[*k*][*k*]_*C*_[*m*][*l*]_ reflects the positive matching degree of correct predictions, and *C*_[*l*][*k*]_*C*_[*k*][*m*]_ eliminates deviation introduced by cross category misclassification, collectively characterizing the overall consistency between true BIO annotations and model outputs.

AUPRC was computed specifically for the B class using a one-versus-rest strategy, in which residues annotated as B were treated as positive samples and residues annotated as O or I were treated as negative samples. Because cleavage site residues represent only a small fraction of all sequence positions, the resulting residue-level classification task is highly imbalanced. Under such conditions, AUPRC provides a more informative assessment than accuracy or AUROC, as it emphasizes the trade-off between precision and recall for the minority class rather than overall classification performance. Consequently, AUPRC is particularly suitable for evaluating rare-event detection tasks, such as functional site identification in biological sequence annotation.^29^

### Quantification and statistical analysis

This study is a computational modeling study and does not involve biological or technical replicates, experimental groups, or hypothesis testing. Therefore, no conventional statistical tests, such as t-tests, analysis of variance, or nonparametric hypothesis tests, were performed, and no *p* values, confidence intervals, standard errors, or standard deviations were calculated.

All model training, evaluation, and metric calculations were performed using Python (v3.11). Model implementation and training were carried out using PyTorch, while metric calculations were performed using scikit-learn. Classification performance was quantitatively evaluated using AUROC, AUPRC, ACC, precision, recall, F1-score, and MCC. The number of samples in each dataset and the corresponding data partitioning procedures are described in the method details and the relevant figure legends and tables.

## Supplemental information

Table S1. Taxonomic distribution of neuropeptide precursor species across different datasets
